# *In Vitro* Modeling of Bile Acid Processing by the Human Fecal Microbiota

**DOI:** 10.3389/fmicb.2018.01153

**Published:** 2018-06-05

**Authors:** Glynn Martin, Sofia Kolida, Julian R. Marchesi, Elizabeth Want, James E. Sidaway, Jonathan R. Swann

**Affiliations:** ^1^Department of Food and Nutritional Sciences, University of Reading, Reading, United Kingdom; ^2^OptiBiotix Health PLC, Innovation Centre, York, United Kingdom; ^3^Division of Integrative Systems Medicine and Digestive Diseases, Imperial College London, London, United Kingdom; ^4^Centre for Digestive and Gut Health, Imperial College London, London, United Kingdom; ^5^School of Biosciences, Cardiff University, Cardiff, United Kingdom; ^6^Phenotox Ltd., Macclesfield, United Kingdom

**Keywords:** metabonomics, metataxonomics, bile acid, microbiota, microbiome, deconjugation, dehydroxylation

## Abstract

Bile acids, the products of concerted host and gut bacterial metabolism, have important signaling functions within the mammalian metabolic system and a key role in digestion. Given the complexity of the mega-variate bacterial community residing in the gastrointestinal tract, studying associations between individual bacterial genera and bile acid processing remains a challenge. Here, we present a novel *in vitro* approach to determine the bacterial genera associated with the metabolism of different primary bile acids and their potential to contribute to inter-individual variation in this processing. Anaerobic, pH-controlled batch cultures were inoculated with human fecal microbiota and treated with individual conjugated primary bile acids (500 μg/ml) to serve as the sole substrate for 24 h. Samples were collected throughout the experiment (0, 5, 10, and 24 h) and the bacterial composition was determined by 16S rRNA gene sequencing and the bile acid signatures were characterized using a targeted ultra-performance liquid chromatography-mass spectrometry (UPLC-MS) approach. Data fusion techniques were used to identify statistical bacterial-metabolic linkages. An increase in gut bacteria associated bile acids was observed over 24 h with variation in the rate of bile acid metabolism across the volunteers (*n* = 7). Correlation analysis identified a significant association between the *Gemmiger* genus and the deconjugation of glycine conjugated bile acids while the deconjugation of taurocholic acid was associated with bacteria from the *Eubacterium* and *Ruminococcus* genera. A positive correlation between *Dorea* and deoxycholic acid production suggest a potential role for this genus in cholic acid dehydroxylation. A slower deconjugation of taurocholic acid was observed in individuals with a greater abundance of *Parasutterella* and *Akkermansia*. This work demonstrates the utility of integrating compositional (metataxonomics) and functional (metabonomics) systems biology approaches, coupled to *in vitro* model systems, to study the biochemical capabilities of bacteria within complex ecosystems. Characterizing the dynamic interactions between the gut microbiota and the bile acid pool enables a greater understanding of how variation in the gut microbiota influences host bile acid signatures, their associated functions and their implications for health.

## Introduction

Bile acids are a classical example of trans-genomic metabolites arising from the combinatorial metabolism of the host genome and the gut microbiome. Primary bile acids, cholic acid (CA) and chenodeoxycholic acid (CDCA), are synthesized in the liver from cholesterol and subsequently conjugated with taurine or glycine residues, creating amphipathic molecules that are stored in the gallbladder. Upon ingestion of a meal bile acids are secreted from the gallbladder into the duodenum. Here, they act as detergents emulsifying fats and reducing the luminal pH to allow pancreatic enzymes to digest carbohydrates and proteins. The majority of bile acids (95–99%) are reabsorbed in the jejunum in their conjugated form and re-enter the enterohepatic circulation (Roberts et al., 2002). The remaining 1–5% enters the ileum and colon (~200–800 mg daily in humans) where they are modified by the gut microbiota. Enzymes encoded in the gut microbiome, such as bile salt hydrolases (BSH) and various dehydroxylases, structurally modify these bile acids, increasing the diversity of the overall pool. Firstly, BSHs cleave the amide bond between the amino acid residue (glycine or taurine) and their bile acid moiety. There are various different BSH enzymes expressed across the microbiota (Jones et al., 2008) and the full impact of BSH diversity has yet to be fully explored. One study observed an overall reduction in bacterial BSH enzyme diversity was associated with an increase in weight gain (Joyce et al., 2014). Once deconjugated the steroid nucleus of the bile acid can undergo further biotransformation. Dehydroxylases (7α or 12α) remove hydroxyl groups from the bile acid forming the secondary bile acids, deoxycholic acid (DCA) from CA and lithocholic acid (LCA) from CDCA. Gut bacterial enzymes can also modify bile acids through various other reactions including epimerization, oxidation, and esterification. Modified bile acids are either excreted in the feces or reabsorbed into the enterohepatic circulation for re-processing in the liver. In the liver, bile acids are re-conjugated with taurine or glycine or are sulfated before secretion into the bile. These combined activities between the microbiota and the host result in a diverse circulating bile acid pool.

Variation in the functionality of the microbiome across individuals can affect these processes with downstream effects on the composition of the bile acid pool. Functional variation is noteworthy because bile acids have an important role in regulating the metabolism of the host. Bile acid moieties, arising from gut bacterial processing such as the secondary and unconjugated primary bile acids, are functional agonists for nuclear receptors found in the liver, heart, intestine, kidneys, and adipose tissue (Forman et al., 1995; Parks et al., 1999; Kim et al., 2007; Björkholm et al., 2009; Xiao et al., 2014). The nuclear receptor farnesoid X receptor (FXR) is activated by CDCA and to a lesser extent than CA, LCA, and DCA, while the plasma membrane bound receptor TGR5 has a high affinity for LCA and its conjugates (Li and Chiang, 2014). These receptors regulate the synthesis, uptake, transportation, and detoxification of bile acids (Chiang, 2009; Fiorucci et al., 2009) as well as the regulation of glucose, cholesterol and triacylglyceride concentrations in plasma and energy metabolism in muscles (Watanabe et al., 2006; Porez et al., 2012). Secondary bile acids can also regulate the expression of xenobiotic enzymes and transporters in the liver, intestine, and kidney *via* pregnane X receptor (PXR) and constitutive androstane receptor (CAR) (Xie et al., 2001; Björkholm et al., 2009). Variation in the bile acid pool may therefore also contribute to individual differences in drug efficacy and responses. We have previously demonstrated the importance of the microbiota in shaping the composition and signaling potential of the bile acid pool (Swann et al., 2011). As bile acids have detergent properties they can also impact on gut bacterial community structure by disrupting bacterial cell membranes, inducing DNA damage and modifying protein structure (Kandell and Bernstein, 1991; Bernstein et al., 1999; Begley et al., 2005; Devkota et al., 2012). The detergent properties of the secondary bile acid DCA are 10 fold greater than its precursor, CA (Kurdi et al., 2006) suggesting that this bacterial modification enhances the antimicrobial activity of the compound. Chemical exchange between gut bacteria and the host through bile acid processing can therefore impact on host health and modulate the community structure of the intestinal microbiome. Characterizing the dynamic interactions between the gut microbiota and the bile acid pool, at the genus level, enables a greater understanding of how variation in the gut microbiota influences host bile acid signatures, their associated functions and their implications for health. In this study, the bacterial processing of conjugated primary bile acids by the human fecal microbiota has been modeled *in vitro*. Bacterial profiles were determined by 16S rRNA gene sequencing analysis and the bacterial metabolism of bile acids was studied over time using an ultra-performance liquid chromatography-mass spectrometry (UPLC-MS) approach.

## Results

### Baseline variation in the bacterial and bile acid profiles of human feces

**Figure 1:**
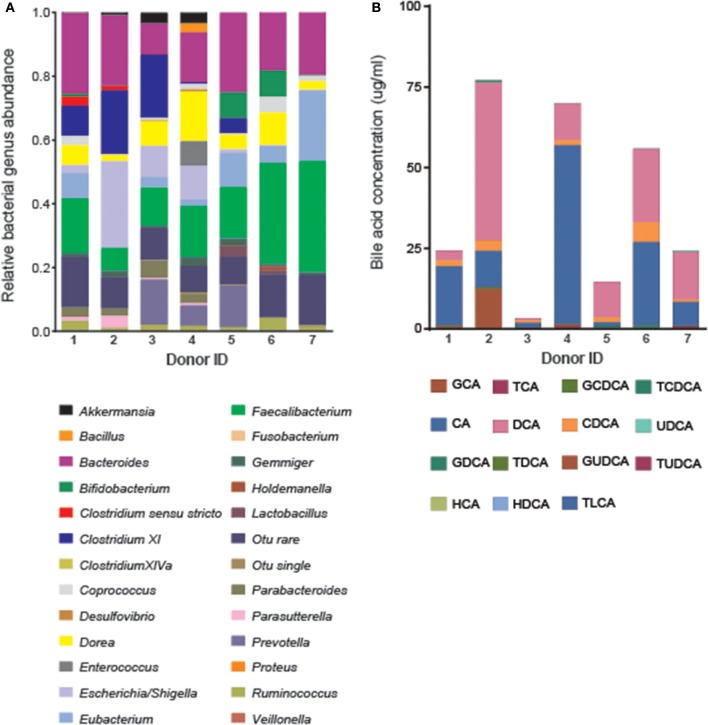
Natural variation in fecal bile acid compositions and bacterial genera across healthy donors before treatment (*n* = 7). **(A)** Fecal bacterial genus abundance was determined by Mothur analysis of the V1-V3 16S rRNA region of all fecal bacteria sequenced for each donor. **(B)** Fifteen bile acids moieties were quantified from the inoculated batch culture medium using UPLC-MS.

Metataxonomic analysis using the V1–V3 region of the 16S rRNA gene provided taxonomic identification to genus level (Figure 1B). At baseline, each donor possessed a unique fecal bacterial profile. Alpha diversity, measured by Chao1 index (mean 42.6, 95% confidence interval: 36.8–51.5) and inverse Simpson diversity index (mean 7.9, 95% confidence interval: 5.4–11.1) (Figure S2), were comparable across the vessels at baseline. No significant differences were found in the total bacterial cell counts using fluorescent *in situ* hybridization (FISH) analysis between donors or treatments at each time point, but a reduction in bacterial numbers (mean decrease of 0.299 log units ± 0.06) was observed across all samples over 24 h (Figure S3).

Bile acid profiles for all seven donors were dominated by the unconjugated primary bile acids CA and CDCA and the unconjugated secondary bile acid DCA at baseline (Figure 1). CA accounted for the majority of the bile acid signature for donors 1 (74.9%), 3 (41.4%), and 4 (79%) while DCA dominated the profiles of donors 2 (63.2%), 5 (74.9%), and 7 (60.2%). Traces of conjugated and unconjugated primary and secondary bile acids were also detected. This included a mean abundance of GCA (4.6 ± 5.4%), GCDCA (1.5 ± 2.1%), TCDCA (0.94 ± 1.3%), TCA (0.65 ± 0.67%), ursodeoxycholic acid (UDCA) (0.39 ± 0.77%), glycodeoxycholic acid (GDCA) (0.09 ± 0.22%), hyodeoxycholic acid (HDCA) (0.07 ± 0.09%), taurolithocholic acid (TLCA) (0.01 ± 0.01%), taurodeoxycholic acid (TDCA) (0.003 ± 0.006%), and tauroursodeoxycholic acid (TUDCA) (0.002 ± 0.003%). No traces of glycoursodeoxycholic acid (GUDCA) or hyocholic acid (HCA) were observed.

### Characterizing the bacterial processing of primary conjugated bile acids

**Figure 2:**
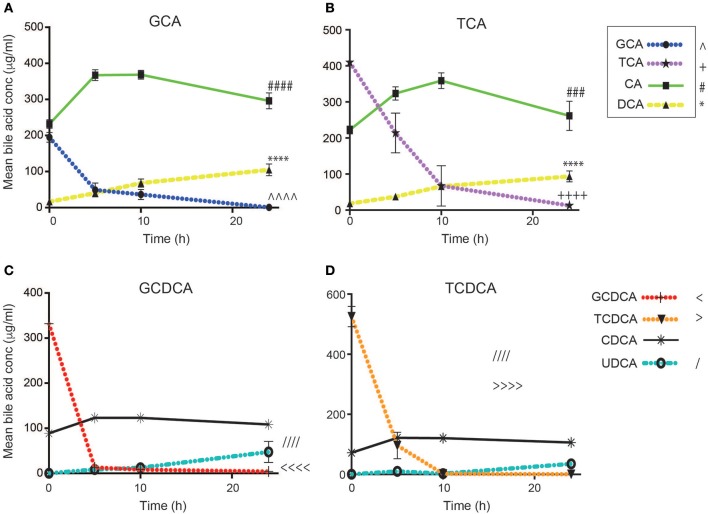
Bile acid concentrations over 24 h following the addition of **(A)** glycocholic acid (GCA), **(B)** taurocholic acid (TCA), **(C)** glycochenodeoxycholic acid (GCDCA), and **(D)** taurochenodeoxycholic acid (TCDCA) to human fecal batch cultures (*n* = 7). Values are mean expressed in μg/ml ± SD. Significant time-dependent differences were observed in the concentrations of the individual bile acids using a one-way ANOVA. **(A)** ^∧∧∧∧^GCA *p* < 0.0001, ^####^CA *p* < 0.0001, ^****^ DCA *p* < 0.001. **(B)** ^++++^TCA *p* < 0.0001, ^###^CA *p* = 0.004, ^****^DCA *p* < 0.001. **(C)** ^< < < < ^GCDCA *p* < 0.0001, ^////^CDCA *p* < 0.0001. **(D)** ^>>>>^TCDCA *p* < 0.0001, ^////^CDCA *p* < 0.0001. CA, cholic acid; DCA, deoxycholic acid; UDCA, ursodeoxycholic acid.

Bile acid profiles were measured in supernatants collected over 24 h from each fecal culture using UPLC-MS and quantified against individual bile acid standards (Figure 2). Bile acids were metabolized by the fecal microbiota and their metabolism was dependent upon the bile acid dosed. This bacterial processing was immediate and even in the baseline samples (0 h), collected shortly after the addition of the bile acid, the concentration of the dosed bile acid was notably reduced. This was most evident in the glycine-conjugated bile acids. For GCA, the amount measured in the baseline sample collected immediately after the addition of the bile acid (500 μg/ml) was 194.9 ± 32.6 μg/ml. At this sampling point, CA was present at 231.7 ± 29.2 μg/ml. By the 10 h sampling point, GCA was reduced to 36.5 ± 27.5 μg/ml (*p* = 0.0012). For GCDCA, the baseline concentration was reduced to 332.3 ± 26 μg/ml and further reduced to 8 ± 4.8 μg/ml over the first 10 h (*p* = 0.0001). In contrast, the taurine-conjugated bile acids were not metabolized to same extent in the baseline samples (TCA, 409.7 ± 30.2 μg/ml; TCDCA, 525.4 ± 82.3 μg/ml). However, all primary conjugated bile acids were found to be deconjugated 10 h post dosing with the TCA and TCDCA concentrations decreasing to 53.6 ± 137.6 μg/ml (*p* = 0.0017) and 2.4 ± 1.2 μg/ml (*p* < 0.0001) respectively, over the first 10 h. The greater abundance of glycine-conjugated bile acids in the human gut compared to those conjugated with taurine (~2.5–3.5 G:T ratio) may underlie these observations.

Accordingly, CA increased in the supernatants of the GCA (231.7 ± 29.2 μg/ml to 368.4 ± 29.6 μg/ml; *p* < 0.0001) and TCA (222.2 ± 26.9 μg/ml to 358.9 ± 67.5 μg/ml; *p* = 0.0005) supplemented vessels while CDCA increased in the GCDCA (88.7 ± 12.4 μg/ml to 123.2 ± 8.4 μg/ml; *p* = 0.0006) and TCDCA (72.1 ± 13.5 μg/ml to 120.5 ± 7.4 μg/ml; *p* = 0.0001) supplemented vessels over the first 10 h. A fall in CA concentrations was observed after 10 h in the GCA and TCA supplemented vessels. This occurred in parallel to an increase in the dehydroxylated form of CA, the secondary bile acid DCA. DCA concentrations rose from 68.2 ± 27.2 μg/ml to 104.6 ± 40.7 μg/ml (*p* = 0.0117) from 10 to 24 h in vessels treated with GCA and 66.1 ± 22.5 μg/ml to 93.7 ± 36.9 μg/ml (*p* = 0.02) in those vessels supplemented with TCA (Figures 2A,B). CDCA concentration remained consistent from 10 to 24 h but a progressive increase in its bacterial-formed epimer, UDCA was observed over 24 h. UDCA concentrations rose from 0.3 ± 0.3 μg/ml at 0 h to 47.5 ± 40.7 μg/ml at 24 h (*p* = 0.1362, *n.s*.) in the GCDCA treated vessels and from 0.7 ± 0.7 μg/ml to 35.2 ± 16 μg/ml (*p* = 0.0781, *n.s*.) in the TCDCA treated vessels. It was not possible to measure LCA, the dehydroxylated form of CDCA, with this approach.

### Illuminating the bacterial genera associated with bile acid metabolism

**Figure 3:**
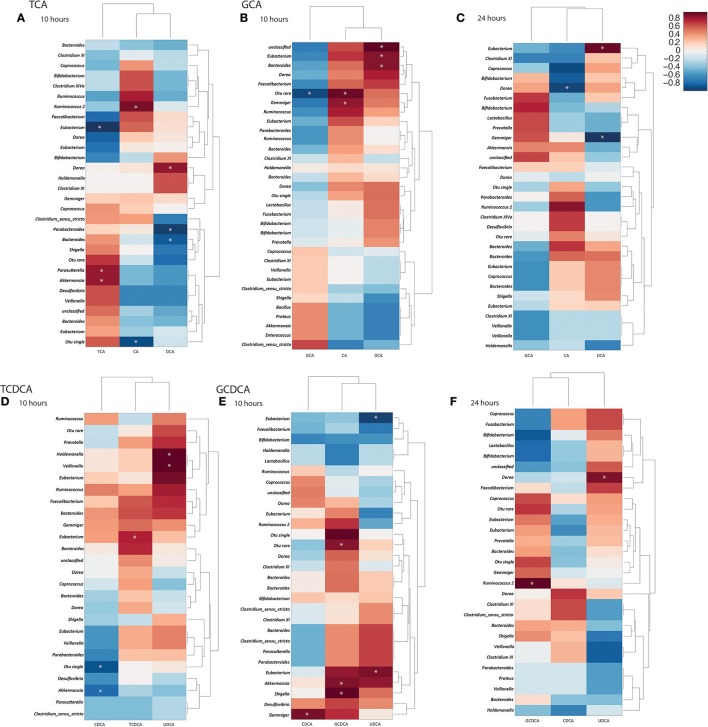
Correlation matrices constructed from bile acid moieties and fecal bacterial genera at 10 and 24 h following bile acid supplementation. **(A)** 10 h post taurocholic acid (TCA); **(B)** 10 h and **(C)** 24 h post glycocholic acid (GCA); **(D)** 10 h post taurochenodeoxycholic acid (TCDCA); **(E)** 10 h and **(F)** 24 h post glycochenodeoxycholic acid (GCDCA) supplementation. Correlation coefficients presented as heat maps with dendrograms indicating relatedness between variables. ^*^Significant correlations (*p* < 0.05). CA, cholic acid; CDCA, chenodeoxycholic acid; DCA, deoxycholic acid; UDCA, ursodeoxycholic acid.

Spearman correlation analysis between the quantified bile acid profiles and the bacterial profiles allowed statistical linkages between bacterial genera and bile acids to be identified at the 10 and 24 h time points independently (Figure 3). A dendrogram was also constructed to indicate relatedness between the variables. Significant associations were found between several bacterial genera and bile acids. At 10 h the majority of primary conjugated bile acids had been deconjugated. Positive correlations were found at 10 h between TCA abundance and *Akkermansia* (*r* = 0.8) and *Parasutterella* (*r* = 0.8). Bacteria from the genus *Eubacterium* were negatively correlated with TCA (*r* = −0.93) at this time point. CA concentrations were positively correlated with *Ruminococcus 2* (*r* = 0.86) at 10 h in the TCA treated cultures. The secondary bile acid, DCA was positively correlated with bacteria from the genus *Dorea* (*r* = 0.81) and negatively correlated with those from the genera *Parabacteroides* (*r* = −0.9) and *Bacteroides* (*r* = −0.8). At 24 h, there were no statistically significant correlations in the TCA supplemented vessels. For GCA supplemented cultures, significant correlations were observed with CA and DCA. At 10 h, CA had positive correlations with *Gemmiger* (*r* = 0.78), whilst DCA positively correlated with *Bacteroides* (*r* = 0.8) and *Eubacterium* (*r* = 0.8). At 24 h, there were negative correlations between *Dorea* and CA (*r* = −0.8). DCA had a positive correlation with *Eubacterium* (*r* = 0.82) and a negative correlation with *Gemmiger* (*r* = −0.85).

Significant correlations were identified between bacterial genera and bile acids in models receiving GCDCA. The genus *Shigella* (*r* = 0.87) was positively correlated with GCDCA at 10 h while the genus *Gemmiger* (*r* = 0.8) was positively correlated with CDCA at this timepoint. At 24 h, GCDCA was strongly positively correlated with *Ruminococcus 2* (*r* = 0.97) while UDCA was positively correlated with *Dorea* (*r* = 0.9). In the models supplemented with TCDCA a positive correlation between *Eubacterium* (*r* = 0.8) and TCDCA was seen at 10 h. This was in addition to a negative correlation between CDCA and *Akkermansia* (*r* = −0.8) and strong correlations between UDCA and *Holdemanella* (*r* = 1) and *Veillonella* (*r* = 1).

### Inter-individual variation in the rate of bacterial bile acid metabolism

**Figure 4:**
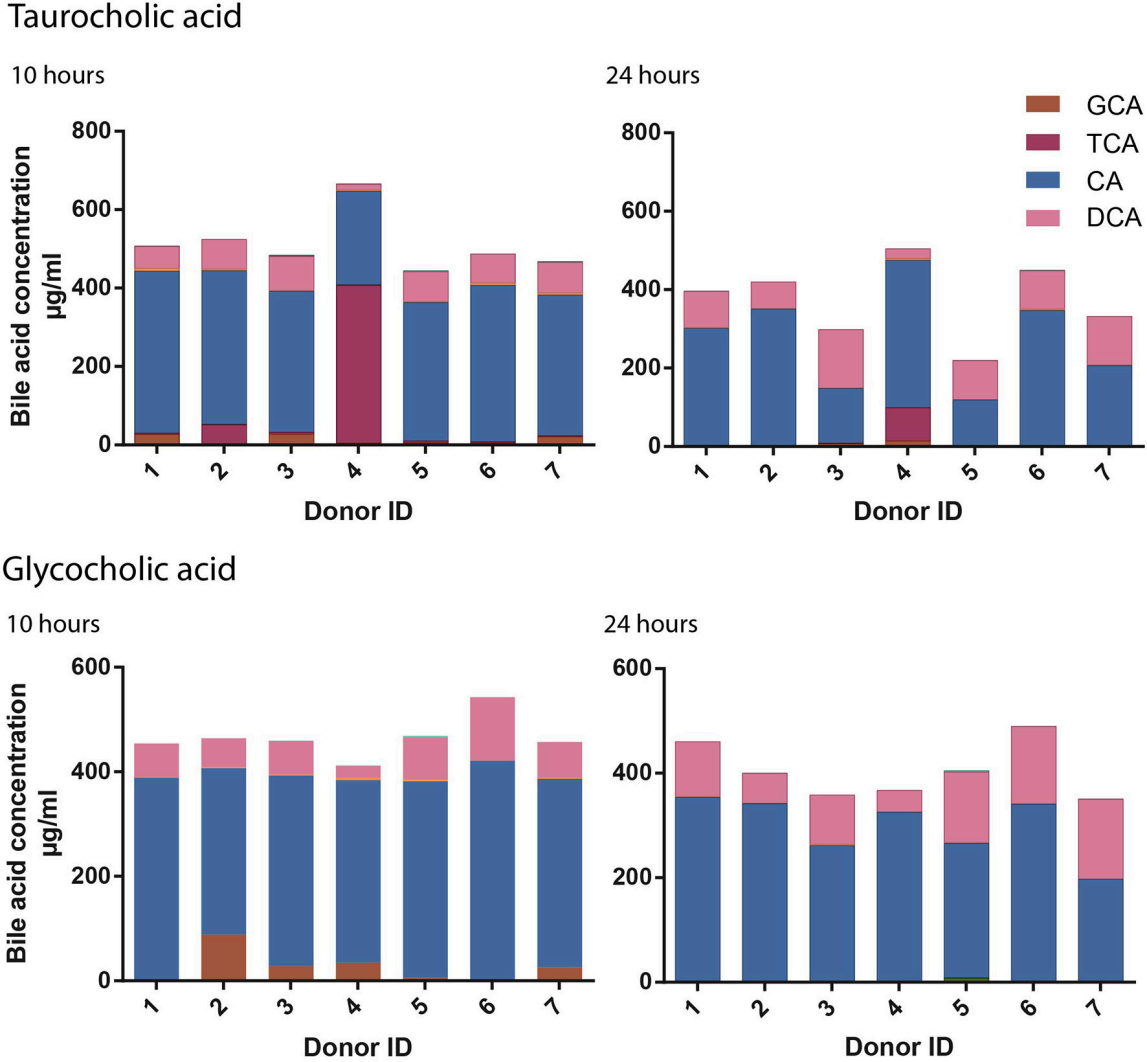
Inter-individual variation in cholate conjugated metabolism. Fifteen bile acid moiety concentrations were quantified by UPLC-MS for each donor (*n* = 7) at 0, 5, 10, and 24 h. The rate of taurocholic acid (TCA) and glycocholic acid (GCA) deconjugation was slower for Donor 4 and Donor 2 respectively at 10 and 24 h post treatment with primary conjugated cholic acid moieties.

Differences in the bile acid moiety concentrations, across the time course, infer that bile acid metabolism occurs at a unique rate across the individual donors. A markedly reduced rate of TCA deconjugation was seen in Donor 4 compared to the other donors over the first 10 h (Figure 4). In this donor the concentration of TCA remained at 402.1 μg/ml at 10 h while being significantly reduced at this time point in the remaining donors (mean 13.3 ± 19.2 μg/ml). Principal components analysis (PCA) was used to assess the variance of the bacterial profiles of the donors throughout the study (Figure S4). At 10 h the bacterial profile of Donor 4 was notably different from the other donors with high proportions of the bacteria from the genera; *Parabacteroides, Bacteroides, Clostridium XIVa Parasutterella* and *Akkermansia*. By 24 h, once TCA metabolism had begun, the bacterial profile of Donor 4 was similar in composition to the other six donors.

A similar, albeit less pronounced, difference in deconjugation was observed across the donors following GCA supplementation. However, with GCA it was Donor 2 that had a slower rate of deconjugation with 88.1 μg/ml of GCA remaining at 10 h compared to 23.5 ± 10.6 μg/ml in the remaining six donors including Donor 4 (34.6 μg/ml).

**Figure 5:**
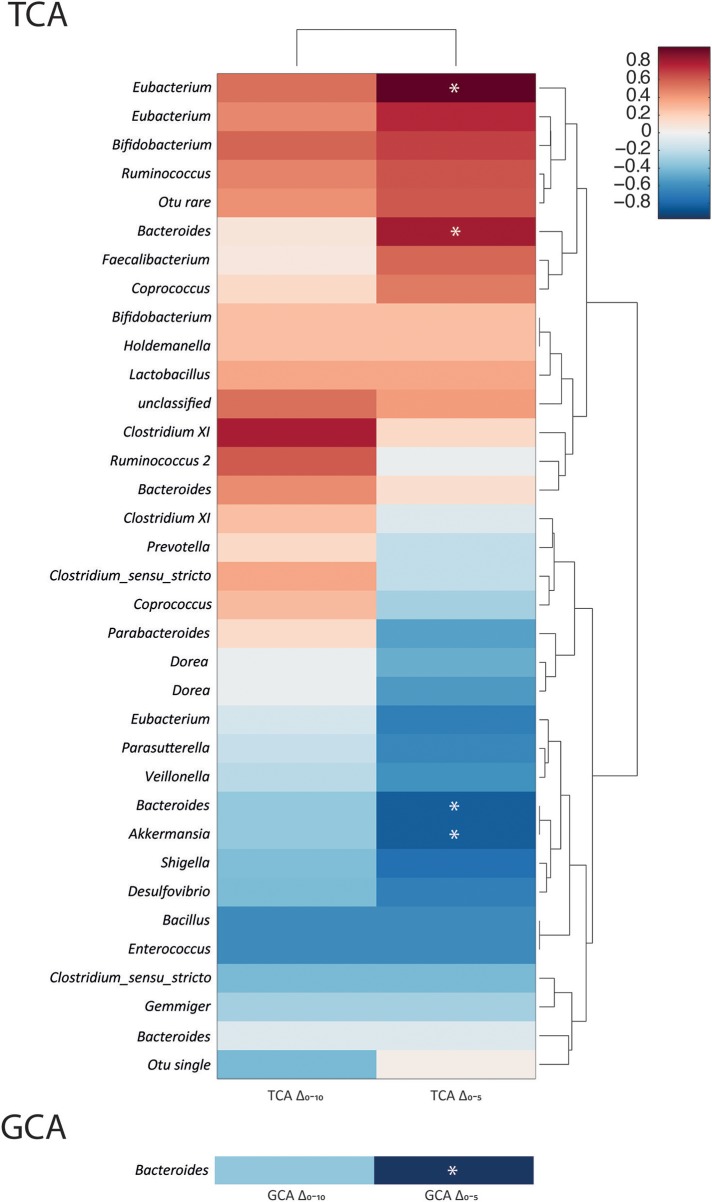
Taurocholic acid deconjugation velocity is positively associated with the *Eubacterium* and *Bacteroides* genera. Correlation coefficients between the bacterial genera present in the batch cultures at baseline and the change in TCA concentrations from 0 to 5 h (ΔTCA_T5_) and 0 to 10 h (ΔTCA_T10_) and GCA concentrations from 0 to 5 h (ΔGCA_T5_) and 0 to 10 h (ΔGCA_T10_). Data presented as heat maps with dendograms indicating relatedness between variables. ^*^Significant correlations (*p* < 0.05).

To investigate the bacterial genera that influence the deconjugation rate of TCA and GCA, correlation analyses were performed. Baseline bacterial profiles were compared to the concentration changes in GCA and TCA between 0 and 5 h (ΔGCA_T5_ and ΔTCA_T5_) and 0 and 10 h (ΔGCA_T10_ and ΔTCA_T10_) (Figure 5). Significant positive correlations were found between the abundance of *Eubacterium* (*r* = 0.96) and *Bacteroides* (*r* = 0.8) at baseline and TCA disappearance over the first 5 h. Negative correlations were observed between *Bacteroides* (*r* = −0.82) and *Akkermansia* abundance (*r* = −0.82) and TCA deconjugation over the first 5 h suggesting that less TCA was deconjugated in vessels containing more bacteria from these genera. The only significant correlation with GCA disappearance over the first 5 h was a negative association with *Bacteroides* (*r* = −0.8). No statistical associations were found for ΔGCA_T10_ and ΔTCA_T10_.

## Discussion

In this study, we demonstrate the utility of using a pH controlled, anaerobic fecal batch culture system to model the bacterial metabolism of individual primary conjugated bile acids. The UPLC-MS results confirm that in an anaerobic fecal batch culture environment, bile acids undergo the same metabolic processes as those occurring *in vivo* (Frankel, 1936; Midtvedt, 1974; Hirano et al., 1981; Fadden et al., 1997). These observations were similar across the majority of donors indicating a consistency in the metabolic performance of the individual donor microbiomes. A potential limitation of this model system is the unknown variation in bacterial succession across the donors in the fermentation vessels. Such variation could affect the reproducibility of the results over extended time periods. However, over the timeframe studied here (0–24 h), which is relevant to intestinal transit times, this variation was not apparent. Combining this model system with an integrated bacterial and bile acid profiling approach has enabled specific bacterial-bile acid associations to be resolved for further investigation. In some cases, these observations can be supported by the genomic content of the bacteria.

Variation was observed across the bacterial and bile acid profiles of all donors at baseline with the bile acid signatures dominated by either unconjugated primary bile acids (CA, CDCA) or the unconjugated secondary bile acid DCA. Greater abundance of fecal DCA in certain donors may result from the increased bacterial dehydroxylation of CA or reduced absorption of this bile acid from the gut of these individuals. Alternatively, this could reflect differences in dietary patterns across the donors with previous studies showing that diets high in animal fat modify the gut microbiota and increase DCA excretion (Yatsunenko et al., 2012; David et al., 2014). High fecal excretion of DCA has been previously linked to obesity and an increased risk of colorectal cancer and hepatic cirrhosis (Bernstein et al., 2005; Kakiyama et al., 2013).

Correlation analysis identified statistical linkages between 15 bacterial OTUs and individual bile acids. These belonged to the *Bacteroidetes, Firmicutes, Proteobacteria*, and *Verrucomicrobia* phyla. A positive association was found between the *Gemmiger* genus and CA concentration 10 h after GCA supplementation. In this case, *Gemmiger* abundance increased following the cleavage of glycine from GCA. Consistently, the *Rumminococcaceae* family, within which *Gemmiger* resides, has been shown to possess a bile salt hydroxylase that acts on both glycine and taurine conjugated bile acids (Jones et al., 2008). No associations were observed between the *Gemmiger* genus and the deconjugation of tauro-conjugated bile acids however, a number of BSH enzymes have specificity toward a single bile acid moiety (Jones et al., 2008). Interestingly, the abundance of the *Gemmiger* genus was negatively correlated with DCA abundance at 24 h. DCA is cytotoxic to certain bacterial species and can be used as an antimicrobial agent. Evidence of these antimicrobial properties are seen with the reduction of *Clostridium difficile* infections when DCA is present in sufficient concentrations (Greathouse et al., 2015). From these observations we speculate that bacteria from the genus *Gemmiger* may be susceptible to DCA toxicity and/or that it's abundance is dependent upon the availability of glycine-conjugated bile acids as a substrate. Furthermore, this observation may indicate that this species does not possess 7-α dehydroxylase, the enzyme required to dehydroxylate the C-7 hydroxyl group of CA to produce DCA. Further experiments are necessary to clarify these hypotheses.

Bacterial species contained within the *Lachnospiraceae* cluster were associated with several bile acids across the treatments. The abundance of bacteria from the *Eubacterium* genus was negatively correlated with TCA at 10 h (TCA-supplemented vessels) and positively correlated with DCA at 24 h (GCA-supplemented vessels). This link suggests that *Eubacterium* abundance increases as TCA is deconjugated to CA and CA is dehydroxylated to DCA. Consistently, members of this genus have been shown to act as dehydroxylators (Gérard, 2013) and possess functional BSH genes (Udayappan et al., 2016). Similarly, the role of the *Ruminococcus 2* genus in the deconjugation of TCA is inferred from the positive association to CA and is consistent with the presence of BSH genes in the genome (Gérard, 2013). The positive associations between the *Holdemanella* and *Veillonella* genera and UDCA at 10 h suggest these two genera may have a role in the epimerization of CDCA, although this observation is unique to the substrate TCDCA and genomic data does not exist to support a causal relationship being defined.

Inter-individual variation was observed in the rate of TCA metabolism. Specifically, the deconjugation of TCA was markedly retarded over the first 10 h for Donor 4 while the remaining donors had near-complete deconjugation over this period. The delay in taurine cleavage coincides with a lower abundance of members from the *Gemmiger, Ruminococcus 2, Eubacterium*, and *Faecalibacteria* genera in this donor and an increased abundance of bacteria from the *Parabacteroides* genus. Interestingly, *P. distasonis* has been associated with gut dysbiosis (Dziarski et al., 2016; Jacobs et al., 2016). Correlation analysis studying the baseline bacterial signatures with changes in the bile acid profiles over the first 5 h revealed a positive association between *Eubacterium* and *Bacteroides* abundance and the rate of TCA disappearance. It is possible that the lower abundance of *Gemmiger, Ruminococcus 2, Eubacterium*, and *Faecalibacteria* reduces the BSH enzyme diversity in Donor 4, which may contribute to the retardation of TCA deconjugation in this donor. Variation in the rate of bile acid deconjugation has downstream consequences for secondary bile acid formation and the potential to alter gut bacterial and host metabolism (Theisen et al., 2000; Jones et al., 2008; Bernstein et al., 2009; Swann et al., 2011; Joyce et al., 2014). Reduced efficiency in bile acid deconjugation could lower the rate at which CA and thus DCA is formed in the proximal colon. Colorectal transit time in a healthy individual is highly variable ranging from 14 to 80 h depending on age and gender (Graff et al., 2001; Haase et al., 2016). A delay of deconjugation by 10 h could therefore result in the greater excretion of primary bile acids, or fewer secondary bile acids formed. Bile acids are potent detergents and have a key role facilitating the absorption of dietary lipids, nutrients and lipid-soluble vitamins. Deconjugation reduces the efficiency of bile acids for the emulsification of dietary lipids and micelle formation while CA has greater lipid-emulsification properties than the dihydroxy bile acids, CDCA, and DCA. Such modulations could impact on the efficiency of the enterohepatic circulation for digesting and absorbing lipids. The downstream effect on host metabolic signaling also needs to be considered with the receptors FXR, PXR, CAR, and TGR5 all binding to unconjugated or secondary bile acids to initiate signaling cascades that influence drug and energy metabolism. Given that these signaling pathways regulate CYP450 enzymes, alterations in the bile acid signatures have potential to modulate the metabolism and clearance of drugs (Kliewer and Willson, 2002; Chatterjee et al., 2005). Furthermore, a feedback loop exists between secondary bile acid formation and gut bacterial proliferation. For example, lower concentrations of DCA have been linked to an increase in *C. difficile* infections (Greathouse et al., 2015). The ability of the gut microbiota to deconjugate and subsequently dehydroxylate bile acids into DCA may protect against *C. difficile* proliferation. This may be a key property in defining the effectiveness of fecal bacterial transplants (FMT) in the treatment of *C. difficile* infections and could explain the poor efficacy of certain donors in this treatment. Hence, this rapid *in vitro* approach may help to assess the efficacy of fecal bacterial transplants prior to use.

These findings demonstrate the hypothesis-generating potential of this approach for studying the metabolic capabilities of complex bacterial communities. The use of *16S* rRNA gene sequencing and not full resolution metagenomics is a limitation of the current approach. While the composition of the bacterial communities has been investigated the functional potential encoded in the genomes of the modeled communities remains unknown. This prevents metabolic outcomes from being confidently attributed to specific bacteria. Based on the obtained results, future experiments combining metagenomic and metabonomic approaches in this model system offer great promise for studying metabolic functions in complex communities and identifying the specific contributions of individual bacteria.

## Conclusion

Complex bacterial communities make it difficult to define the contribution of each individual microbe to the overall network. This work demonstrates the utility of using an *in vitro* model of the human fecal microbiota, coupled with integrated systems biology techniques, to elucidate specific bacterial-bile acid associations against a background of complex community interactions. Improving the resolution at which we can study these associations is of great benefit given the importance of the enterohepatic circulation in the regulation of host energy, lipid, glucose, and xenobiotic metabolism.

## Materials and methods

### Closed anaerobic fecal microbiota fermentation

Seven healthy donors, four male and three female aged 22–40 years, who did not have any history of gastrointestinal disorders and had avoided probiotics, prebiotics, and antibiotics for at least 3 months prior to the study, were chosen (Study design illustrated in Figure S1). After obtaining verbal informed consent, a standard questionnaire to collect information regarding the health status, drugs use, clinical anamnesis, and lifestyle was administrated before the donor was ask to provide a fecal sample. The University of Reading ethics committee exempted this study from review because no donors were involved in any intervention and waived the need for written consent due to the fact the samples received were not collected by means of intervention. Fecal slurries were prepared by homogenizing freshly voided human feces (10%, w/v) in phosphate-buffered saline (PBS; 0.1 M, pH 7.4) using a stomacher (model 6041; Seward Scientific, U.K.) at 460 beats/min for 120 s. Each fermentation vessel was inoculated with 6 ml of fecal slurry to 53 ml of basal medium to a final concentration of 10% v/v. All vessels set up in parallel were inoculated with the same fecal sample. Fermentation vessels were maintained under anaerobic conditions, at 37°C, by constant sparging with O_2_-free N_2_. The pH was maintained at pH 6.7 (±0.1) by means of an automated pH controller (Fermac 260; Electrolab, Tewkesbury, U.K.), which added 1 mol/L sodium hydroxide or hydrochloric acid as appropriate. The basal medium contained, per liter, 2 g peptone water (Oxoid Ltd., Basingstoke, U.K.), 2 g yeast extract (Oxoid), 0.01 g sodium chloride (BDH), 0.04 g dipotassium phosphate (BDH), 0.04 g, monopotassium phosphate (BDH), 0.01 g magnesium sulfate heptahydrate (BDH), 0.01 g calcium chloride hexahydrate (BDH), 2 g of sodium bicarbonate (BDH), 2 mL Tween 80 (BDH), 0.05 g haemin (Sigma, Dorset, U.K.), 10 μL vitamin K1 (Sigma), 0.5 g cysteine-hydrochloric acid, and 1 mg resazurin (Sigma).

Five minutes after fecal inoculation, 1 ml of (30 mg/ml) primary conjugated bile acid; taurocholic acid (Sigma Aldrich); glycocholic acid (Sigma Aldrich); taurochenodeoxycholic acid (Sigma Aldrich); or glycochenodeoxycholic acid (Sigma Aldrich); in PBS was added to four of the fermentation vessels to give a final concentration of 500 μg/ ml per vessel with 1 ml of PBS added to the control fermentation vessel. Samples (3 mL) were removed at 0, 5, 10, and 24 h after incubation with the bile acid substrate. These were prepared for analysis by UPLC-MS and 16S rRNA sequencing.

### UPLC-MS analysis of fecal water samples

Fecal water samples were analyzed using UPLC-MS as described by Want et al. (2010). Fecal water samples were prepared by centrifugation at 8,000 *g* for 10 min to remove proteinous matter from the batch culture method. The supernatant from each sample was transferred to a fresh 1.5 ml microcentrifuge tube and stored at −80°C until analysis. Bile acid standards were obtained from Sigma Aldrich (Gillingham, U.K) (TUDCA, TLCA, GDCA, GUDCA, TDCA, UDCA, GCA, LCA, TCDCA, TCA, GCDCA, and DCA) and Steraloids Inc (London U.K) (CDCA, CA, HDCA, and HCA). Bile acid stock solutions (1 mg/ml) were prepared in water: methanol [80:20 (v/v)] solution and used immediately. Water, acetonitrile and methanol were Fluka LC-MS CHROMASOLV, all obtained from Sigma Aldrich (Gillingham, U.K). Calibration curves were created from bile acid stocks by creating nine standards for each of the 16 bile acid moieties as follows; 500, 250, 125, 62.5, 10, 1, 100, 10, and 1 ng/ml. A pooled fecal water sample (QC) was prepared by combining 10 μl of each fecal water sample from the batch culture experiments and was injected 10 times at the start of the run to condition the column and every 10 samples. The purpose of the QC sample was to assess instrument stability and to ensure consistency of peaks throughout the sample batch. Hundred microliter of each fecal water sample was transferred into a 350 μl volume 96 well plate. Samples were analyzed using an Acquity UPLC system connected to a Q-TOF premier mass spectrometer (Waters MS technologies, Ltd, Manchester, UK). The column was a CSH C18 1.7 μM (Waters) UPLC (2.1 × 100 mm), the flow rate was 0.5 ml/min. The run time per sample was 12 min, 8 μl of each sample was injected with mobile phase starting conditions of 15% acetonitrile and 85% water. Acetonitrile concentration was increased linearly over the run whilst water decreased, until a final solvent composition of 85% acetonitrile and 15% water was reached. Samples were analyzed in negative electrospray mode over a scan range of 50–1,000 m/z. Bile acids ionize strongly in negative mode, producing a prominent [M-H]- ion and characteristic fragmentation data. MS conditions were as follows: Capillary voltage 2.4 kV, sample cone 30 V, desolvation temperature 400°C, source temperature 120°C, scan duration 0.2 s, cycle time 0.22 s, and interscan delay 0.02 s.

Data were analyzed through MassLynx software (Waters Corp.). Standard curves were constructed by least-squares linear regression analysis using each individual bile acid moiety peak area as calculated using the ApexTrack2 function vs. the concentration of the standard. TargetLynx application manager in Masslynx software (Waters Corp.) was used to generate intensity (peak area) and concentration using the calibration curve from the bile acid standards, for each bile acid in every sample. Paired *t-*tests and one way ANOVA were performed using GraphPad Prism 6 (GraphPad Software, Inc) and Matlab®.

## 16S rRNA gene sequencing analysis

### DNA extraction

Frozen fecal water samples in 50% v/v glycerol from each culture vessel were defrosted, centrifuged at 8,000 *g* for 1 min and the supernatant was removed. Glycerol was used as a preserving agent for cell viability, while in storage at −80°C. The cells were washed in 1 ml sterile PBS. Cells were resuspended in 0.5 ml TES buffer with the addition of 8 μl lysozyme and 2 μl mutanolysin and incubated for 30 min at 37°C. Proteinase K (10 μl) and RNase (10 μl) was added before incubation at 65°C for 60 min and 10% SDS and further incubation at 65°C for 15 min. This preparation was combined with 620 μl phenol/ chloroform/ water (25:24:1), the microcentrifuge tubes were inverted and centrifuged for 10 min at 1,500 *g*. The aqueous layer was removed from sample, transferred to a microcentrifuge tube and combined with 1 ml of ice cold ethanol. Samples were stored at −20°C overnight. Samples were centrifuged for 30 min at 5,668 *g*, the supernatant was removed and the DNA was left to dry before resuspension in 50 μl H_2_O.

### Amplification of 16S rRNA sequence

DNA samples were amplified for sequencing using a forward and reverse fusion primer. The forward primer was constructed with (5′-3′) the Illumina i5 adapter (AATGATACGGCGACCACCGAGATCTACAC), an 8–10 bp barcode, a primer pad, and the 28F primer (GAGTTTGATCNTGGCTCAG). The reverse fusion primer was constructed with (5′-3′) the Illumina i7 adapter (CAAGCAGAAGACGGCATACGAGAT), an 8–10 bp barcode, a primer pad, and the 519R primer (GTNTTACNGCGGCKGCTG). Primer pads were designed to ensure the primer pad/primer combination had a melting temperature of 63–66°C. Amplifications were performed in 25 μl reactions with Qiagen HotStar *Taq* master mix (Qiagen Inc, Valencia, California), 1 μl of each 5 μM primer, and 1 μl of template. Reactions were performed on ABI Veriti thermocyclers (Applied Biosytems, Carlsbad, California) under the following thermal profile: 95°C for 5 min, 25 cycles of 94°C for 30 s, 54°C for 40 s, 72°C for 1 min, followed by one cycle of 72°C for 10 min and 4°C hold.

Amplification products were visualized with eGels (Life Technologies, Grand Island, New York). Products were pooled equimolar and each pool was size selected in two rounds using Agencourt AMPure XP (BeckmanCoulter, Indianapolis, Indiana) in a 0.7 ration for both rounds. Size selected pools were quantified using the Quibit 2.0 fluorometer (Life Technologies) and loaded on an Illumina MiSeq (Illumina, Inc. San Diego, California) 2 × 300 flow cell at 10 pM.

### Data analysis

Raw sequence data from the Illumina platform was processed using Mothur (Schloss et al., 2009) running on Linux. Using Schloss' SOP for MiSeq we assessed the quality of the data and, pre-processed, assembled paired-end reads, removed chimeras, and aligned to obtain a total number of sequences in each sample. To analyze the bacterial communities of batch culture samples, Mothur clustered the data according to operational taxonomic units (OTUs), at a 97% cut-off. Singletons and any OTUs which were not found more than 10 times in any sample were collated into OTU_singletons and OTU_rare phylotypes, respectively, to maintain normalization and to minimize artifacts. The shared OTU data from Mothur (taxonomic data) was analyzed using a combination of statistical programs R-script, stamp (Parks and Beiko, 2010). The two methods used in this study to compare the similarity between each sample were Jaccard's index of similarity and the Bray–Curtis distance measure (measure of dissimilarity) (Lozupone and Knight, 2005; Wang et al., 2013). Weighted Unifrac distance matrices were analyzed in R using non-metric multidimensional scaling ordination and the shared OTU file was used to determine the number of times that an OTU was observed in multiple samples and for multivariate analysis in R. OTU taxonomies (from phylum to genus) were determined using the classifier command in Mothur to generate the RDP taxonomy (Wang et al., 2007). A closed reference approach was used to obtain genus level taxonomic classifications. The OTUs identified were compared to the RDP database of 12,681 bacterial and 531 archael 16S rRNA gene sequences with associated taxonomy. Once this information has been obtained the genome sequence of the genera were studied for genes identified as either a bile salt hydrolase or choloylglycine hydrolase. Alpha and beta indices were calculated from these datasets with Mothur and R using the Vegan package.

### Fluorescent *in situ* hybridization

Aliquots (500 μL) of batch culture samples were fixed in ice-cold 4% (w/v) paraformaldehyde for 4 h at 4°C. These samples were centrifuged at 13,000 *g* for 5 min and washed twice in 1 mL of sterile PBS. The cells were pelleted by centrifugation and resuspended in 150 μL of sterile PBS, to which 150 μL of ethanol was added. The samples were vortexed and stored at −20°C until used in hybridizations.

For the hybridizations, each sample was diluted 1 in 250 to stop clumping of bacteria invalidating microscopy cell counts. Twenty microliter of each sample was pipetted onto Teflon- and poly-l-lysine-coated, six-well (10 mm diameter each) slides (Tekdon Inc., Myakka City, FL). The samples were dried onto the slides at 46°C for 15 min and afterwards dehydrated in an alcohol series (50, 80, and 96%, 3 min each). The ethanol was allowed to evaporate from the slides before the probes were applied to the samples. A probe/hybridization buffer mixture (5 μL of a 50 ng μL^−1^ stock of EUB338 probe plus 45 μL of hybridization buffer) was applied to the surface of each well. Hybridizations were performed for 4 h in an ISO20 oven (Grant Boekel). For the washing step, slides were placed in 50 mL of wash buffer containing 20 μL of 4′,6-diamidino-2-phenylindole dihydrochloride (DAPI; 50 ng μL^−1^; Sigma) for 15 min. They were then briefly washed (2–3 s) in ice-cold water and dried under a stream of compressed air. Five microliters of antifade reagent (polyvinyl alcohol mounting medium with DABCO™ antifading; Sigma) was added to each well and a coverslip was applied. Slides were stored in the dark at 4°C (for a maximum of 3 days) until cells were counted under a Nikon E400 Eclipse microscope. DAPI slides were visualized with the aid of a DM 400 filter and probe slides with the aid of a DM 575 filter. Numbers of specific bacteria and DAPI-stained entities (used to count total bacteria) were determined using the following equation: 0.8 × ACC × 6732.42 × 50 × DF, where DF is the dilution factor and ACC is the average cell count of 15 fields of view. The figure 6732.42 refers to the area of the well divided by the area of the field of view and the factor 50 takes the cell count back to per mL of sample.

### Data integration

Spearman correlation analysis was performed at each sampling point independently correlating the quantified bile acids with the bacterial abundances across all donors. The number of bacteria and bile acids included in the test differed across sampling points and with the different test bile acids. Significant associations were identified when *p* < 0.05.

## Author contributions

All authors listed, have made substantial, direct and intellectual contribution to the work, and approved it for publication.

### Conflict of interest statement

The authors declare that the research was conducted in the absence of any commercial or financial relationships that could be construed as a potential conflict of interest.

## References

[B1] Begley M. Gahan C. G. Hill C. (2005). The interaction between bacteria and bile. FEMS Microbiol. Rev. 29, 625–651. 10.1016/j.femsre.2004.09.003 16102595

[B2] Bernstein H. Bernstein C. Payne C. M. Dvorak K. (2009). Bile acids as endogenous etiologic agents in gastrointestinal cancer. World J. Gastroenterol. 15, 3329–3340. 10.3748/wjg.15.3329 19610133 PMC2712893

[B3] Bernstein H. Bernstein C. Payne C. M. Dvorakova K. Garewal H. (2005). Bile acids as carcinogens in human gastrointestinal cancers. Mutat. Res. 589, 47–65. 10.1016/j.mrrev.2004.08.001 15652226

[B4] Bernstein H. Payne C. M. Bernstein C. Schneider J. Beard S. E. Crowley C. L. (1999). Activation of the promoters of genes associated with DNA damage, oxidative stress, ER stress and protein malfolding by the bile salt, deoxycholate. Toxicol. Lett. 108, 37–46. 10.1016/S0378-4274(99)00113-7 10472808

[B5] Björkholm B. Bok C. M. Lundin A. Rafter J. Hibberd M. L. Pettersson S. (2009). Intestinal microbiota regulate xenobiotic metabolism in the liver. PLoS ONE 4:e6958. 10.1371/journal.pone.0006958 19742318 PMC2734986

[B6] Chatterjee B. Echchgadda I. Song C. S. (2005). Vitamin D receptor regulation of the steroid/bile acid sulfotransferase SULT2A1. Meth. Enzymol. 400, 165–191. 10.1016/S0076-6879(05)00010-8 16399349

[B7] Chiang J. Y. (2009). Bile acids: regulation of synthesis. J. Lipid Res. 50, 1955–1966. 10.1194/jlr.R900010-JLR200 19346330 PMC2739756

[B8] David L. A. Maurice C. F. Carmody R. N. Gootenberg D. B. Button J. E. Wolfe B. E. (2014). Diet rapidly and reproducibly alters the human gut microbiome. Nature 505, 559–563. 10.1038/nature12820 24336217 PMC3957428

[B9] Devkota S. Wang Y. Musch M. W. Leone V. Fehlner-Peach H. Nadimpalli A. (2012). Dietary-fat-induced taurocholic acid promotes pathobiont expansion and colitis in Il10-/- mice. Nature 487, 104–108. 10.1038/nature11225 22722865 PMC3393783

[B10] Dziarski R. Park S. Y. Kashyap D. R. Dowd S. E. Gupta D. (2016). Pglyrp-regulated gut microflora *Prevotella falsenii, Parabacteroides distasonis* and *Bacteroides eggerthii* enhance and *Alistipes finegoldii Attenuates Colitis* in mice. PLoS ONE 11:e0146162. 10.1371/journal.pone.0146162 26727498 PMC4699708

[B11] Fadden K. Hill M. J. Owen R. W. (1997). Effect of fibre on bile acid metabolism by human faecal bacteria in batch and continuous culture. Eur. J. Cancer Prev. 6, 175–194. 9237069

[B12] Fiorucci S. Mencarelli A. Palladino G. Cipriani S. (2009). Bile-acid-activated receptors: targeting TGR5 and farnesoid-X-receptor in lipid and glucose disorders. Trends Pharmacol. Sci. 30, 570–580. 10.1016/j.tips.2009.08.001 19758712

[B13] Forman B. M. Goode E. Chen J. Oro A. E. Bradley D. J. Perlmann T. (1995). Identification of a nuclear receptor that is activated by farnesol metabolites. Cell 81, 687–693. 10.1016/0092-8674(95)90530-8 7774010

[B14] Frankel M. (1936). The biological splitting of conjugated bile acids. Biochem. J. 30, 2111–2116. 10.1042/bj0302111 16746268 PMC1263310

[B15] Gérard P. (2013). Metabolism of cholesterol and bile acids by the gut microbiota. Pathogens 3, 14–24. 10.3390/pathogens3010014 25437605 PMC4235735

[B16] Graff J. Brinch K. Madsen J. L. (2001). Gastrointestinal mean transit times in young and middle-aged healthy subjects. Clin. Physiol. 21, 253–259. 10.1046/j.1365-2281.2001.00308.x 11318834

[B17] Greathouse K. L. Harris C. C. Bultman S. J. (2015). Dysfunctional families: *Clostridium scindens* and secondary bile acids inhibit the growth of *Clostridium difficile*. Cell Metab. 21, 9–10. 10.1016/j.cmet.2014.12.016 25565200 PMC6333312

[B18] Haase A. M. Gregersen T. Christensen L. A. Agnholt J. Dahlerup J. F. Schlageter V. (2016). Regional gastrointestinal transit times in severe ulcerative colitis. Neurogastroenterol. Motil. 28, 217–224. 10.1111/nmo.12713 26729638

[B19] Hirano S. Masuda N. Oda H. Imamura T. (1981). Transformation of bile acids by mixed microbial cultures from human feces and bile acid transforming activities of isolated bacterial strains. Microbiol. Immunol. 25, 271–282. 10.1111/j.1348-0421.1981.tb00029.x 7253965

[B20] Jacobs J. P. Goudarzi M. Singh N. Tong M. McHardy I. H. Ruegger P.. (2016). A disease-associated microbial and metabolomics state in relatives of pediatric inflammatory bowel disease patients. Cell. Mol. Gastroenterol. Hepatol. 2, 750–766. 10.1016/j.jcmgh.2016.06.004 28174747 PMC5247316

[B21] Jones B. V. Begley M. Hill C. Gahan C. G. Marchesi J. R. (2008). Functional and comparative metagenomic analysis of bile salt hydrolase activity in the human gut microbiome. Proc. Natl. Acad. Sci. U.S.A. 105, 13580–13585. 10.1073/pnas.0804437105 18757757 PMC2533232

[B22] Joyce S. A. MacSharry J. Casey P. G. Kinsella M. Murphy E. F. Shanahan F. (2014). Regulation of host weight gain and lipid metabolism by bacterial bile acid modification in the gut. Proc. Natl. Acad. Sci. U.S.A. 111, 7421–7426. 10.1073/pnas.1323599111 24799697 PMC4034235

[B23] Kakiyama G. Pandak W. M. Gillevet P. M. Hylemon P. B. Heuman D. M. Daita K. (2013). Modulation of the fecal bile acid profile by gut microbiota in cirrhosis. J. Hepatol. 58, 949–955. 10.1016/j.jhep.2013.01.003 23333527 PMC3936319

[B24] Kandell R. L. Bernstein C. (1991). Bile salt/acid induction of DNA damage in bacterial and mammalian cells: implications for colon cancer. Nutr. Cancer. 16, 227–238. 10.1080/01635589109514161 1775385

[B25] Kim I. Ahn S. H. Inagaki T. Choi M. Ito S. Guo G. L. (2007). Differential regulation of bile acid homeostasis by the farnesoid X receptor in liver and intestine. J. Lipid Res. 48, 2664–2672. 10.1194/jlr.M700330-JLR200 17720959

[B26] Kliewer S. A. Willson T. M. (2002). Regulation of xenobiotic and bile acid metabolism by the nuclear pregnane X receptor. J. Lipid Res. 43, 359–364. 11893771

[B27] Kurdi P. Kawanishi K. Mizutani K. Yokota A. (2006). Mechanism of growth inhibition by free bile acids in lactobacilli and bifidobacteria. J. Bacteriol. 188, 1979–1986. 10.1128/JB.188.5.1979-1986.2006 16484210 PMC1426545

[B28] Li T. Chiang J. Y. (2014). Bile acid signaling in metabolic disease and drug therapy. Pharmacol. Rev. 66, 948–983. 10.1124/pr.113.008201 25073467 PMC4180336

[B29] Lozupone C. Knight R. (2005). UniFrac: a new phylogenetic method for comparing microbial communities. Appl. Environ. Microbiol. 71, 8228–8235. 10.1128/AEM.71.12.8228-8235.2005 16332807 PMC1317376

[B30] Midtvedt T. (1974). Microbial bile-acid transformation. Am. J. Clin. Nutr. 27, 1341–1347. 10.1093/ajcn/27.11.1341 4217103

[B31] Parks D. H. Beiko R. G. (2010). Identifying biologically relevant differences between metagenomic communities. Bioinformatics 26, 715–721. 10.1093/bioinformatics/btq041 20130030

[B32] Parks D. J. Blanchard S. G. Bledsoe R. K. Chandra G. Consler T. G. Kliewer S. A. (1999). Bile acids: natural ligands for an orphan nuclear receptor. Science 284, 1365–1368. 10.1126/science.284.5418.1365 10334993

[B33] Porez G. Prawitt J. Gross B. Staels B. (2012). Bile acid receptors as targets for the treatment of dyslipidemia and cardiovascular disease. J. Lipid Res. 53, 1723–1737. 10.1194/jlr.R024794 22550135 PMC3413216

[B34] Roberts M. S. Magnusson B. M. Burczynski F. J. Weiss M. (2002). Enterohepatic circulation: physiological, pharmacokinetic and clinical implications. Clin. Pharmacokinet. 41, 751–790. 10.2165/00003088-200241100-00005 12162761

[B35] Schloss P. D. Westcott S. L. Ryabin T. Hall J. R. Hartmann M. Hollister E. B. (2009). Introducing mothur: open-source, platform-independent, community-supported software for describing and comparing microbial communities. Appl. Environ. Microbiol. 75, 7537–7541. 10.1128/AEM.01541-09 19801464 PMC2786419

[B36] Swann J. R. Want E. J. Geier F. M. Spagou K. Wilson I. D. Sidaway J. E. (2011). Systemic gut microbial modulation of bile acid metabolism in host tissue compartments. Proc. Natl. Acad. Sci. U.S.A. 108, 4523–4530. 10.1073/pnas.1006734107 20837534 PMC3063584

[B37] Theisen J. Nehra D. Citron D. Johansson J. Hagen J. A. Crookes P. F. (2000). Suppression of gastric acid secretion in patients with gastroesophageal reflux disease results in gastric bacterial overgrowth and deconjugation of bile acids. J. Gastroint. Surg. 4, 50–54. 10.1016/S1091-255X(00)80032-3 10631362

[B38] Udayappan S. Manneras-Holm L. Chaplin-Scott A. Belzer C. Herrema H. Dallinga-Thie G. M. (2016). Oral treatment with *Eubacterium hallii* improves insulin sensitivity in db/db mice. NPJ Biofilms Microbiomes 2:16009. 10.1038/npjbiofilms.2016.9 28721246 PMC5515273

[B39] Wang J. Qi J. Zhao H. He S. Zhang Y. Wei S. (2013). Metagenomic sequencing reveals microbiota and its functional potential associated with periodontal disease. Sci. Rep. 3:1843. 10.1038/srep01843 23673380 PMC3654486

[B40] Wang Q. Garrity G. M. Tiedje J. M. Cole J. R. (2007). Naive Bayesian classifier for rapid assignment of rRNA sequences into the new bacterial taxonomy. Appl. Environ. Microbiol. 73, 5261–5267. 10.1128/AEM.00062-07 17586664 PMC1950982

[B41] Want E. J. Coen M. Masson P. Keun H. C. Pearce J. T. Reily M. D. (2010). Ultra performance liquid chromatography-mass spectrometry profiling of bile acid metabolites in biofluids: application to experimental toxicology studies. Anal. Chem. 82, 5282–5289. 10.1021/ac1007078 20469835

[B42] Watanabe M. Houten S. M. Mataki C. Christoffolete M. A. Kim B. W. Sato H. (2006). Bile acids induce energy expenditure by promoting intracellular thyroid hormone activation. Nature 439, 484–489. 10.1038/nature04330 16400329

[B43] Xiao L. Zhang Z. Luo X. (2014). Roles of xenobiotic receptors in vascular pathophysiology. Circul. J. 78, 1520–1530. 10.1253/circj.CJ-14-0343 24859622

[B44] Xie W. Radominska-Pandya A. Shi Y. Simon C. M. Nelson M. C. Ong E. S. (2001). An essential role for nuclear receptors SXR/PXR in detoxification of cholestatic bile acids. Proc. Natl. Acad. Sci. U.S.A. 98, 3375–3380. 10.1073/pnas.051014398 11248086 PMC30661

[B45] Yatsunenko T. Rey F. E. Manary M. J. Trehan I. Dominguez-Bello M. G. Contreras M. (2012). Human gut microbiome viewed across age and geography. Nature 486, 222–227. 10.1038/nature11053 22699611 PMC3376388

