# Supplementary material for: In Vitro Modeling of Bile Acid Processing by the Human Fecal Microbiota
Source: Front Microbiol. 2018 Jun 5;9:1153. doi: 10.3389/fmicb.2018.01153 (PMC5996868; doi:10.3389/fmicb.2018.01153)
Supplement: Supplementary file 1 [file Data_Sheet_1.docx]

Supplementary Material

# *In Vitro* Modeling of Bile Acid Processing by the Human Fecal Microbiota

**Glynn Martin, Sofia Kolida, Julian R. Marchesi, Elizabeth Want, James E. Sidaway, Jonathan R. Swann^*^**

*** Correspondence:** Jonathan R. Swann: j.swann@imperial.ac.uk

Supplementary Figures


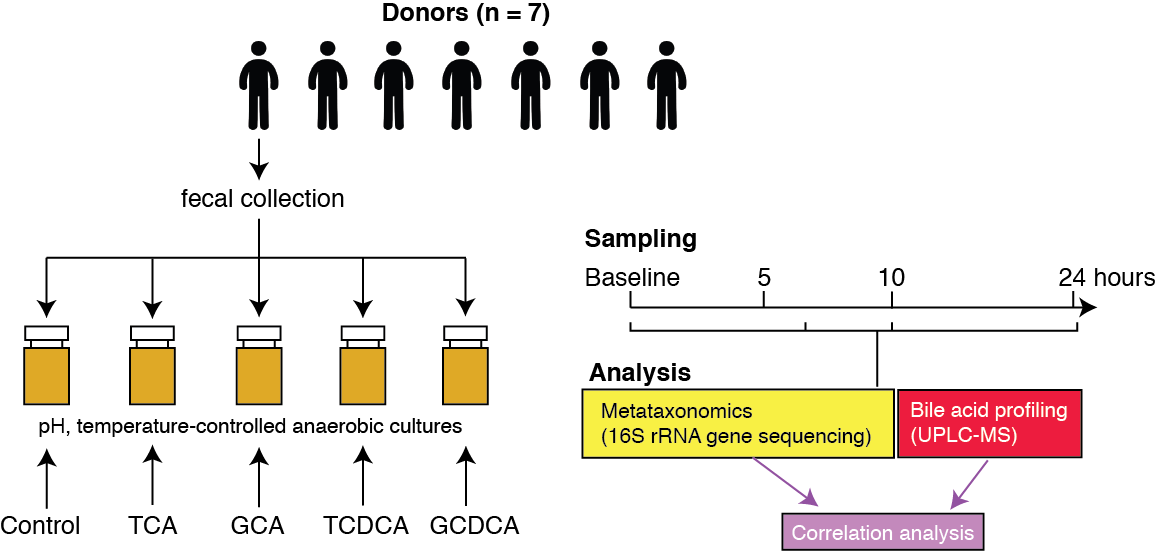


**Figure S1**: Study design. TCA, taurocholic acid; GCA, glycocholic acid; TCDCA, taurochenodeoxycholic acid; GCDCA, glycochenodeoxycholic acid. UPLC-MS, ultra-performance liquid chromatography-mass spectrometry.


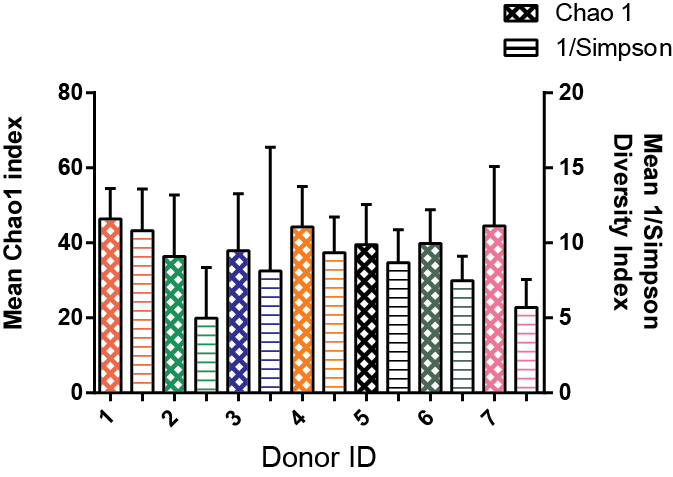


Figure S2: Baseline alpha diversity for each of the seven fecal donors as determined by the mean Chao1 (left Y axis) and inverse Simpson (right Y axis) indices for each of the baseline treatments (*n* = 5). Chao1 and Inverse Simpson indices were determined by Mothur analysis.

Fluorescent in situ hybridisation (FISH) analysis was used to quantify the total number of Eubacteria at each time point across all seven donors after each bile acid treatment. Mean microbial counts for all donors and each treatment group do not vary significantly from each other at each time point. However, a one way ANOVA across the three time points indicates a reduction in microbial populations over 24 hours (p <0.0001). Further multiple comparisons show that total viable microbial differences are seen between zero and ten hours (a reduction of 0.2314 log units) and zero and 24 hours (a reduction of 0.2993 log units), but not between ten and 24 hours (Supplementary Figure S2).


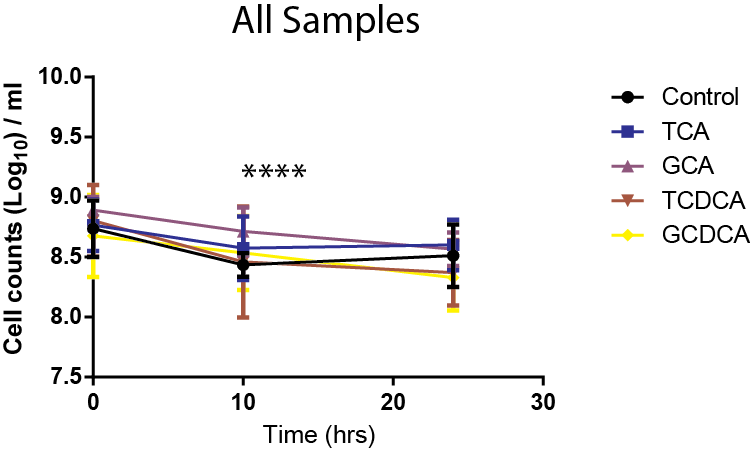


**Figure S3:** A time course for the mean and standard deviation of the total number of viable fecal microbes for all donors for each bile acid treatment over 24 hours. (*n* = 35) Over 24 hours there is a statistical significant decline in microbial counts established by a one-way ANOVA across the three time points (p <0.0001, ****).


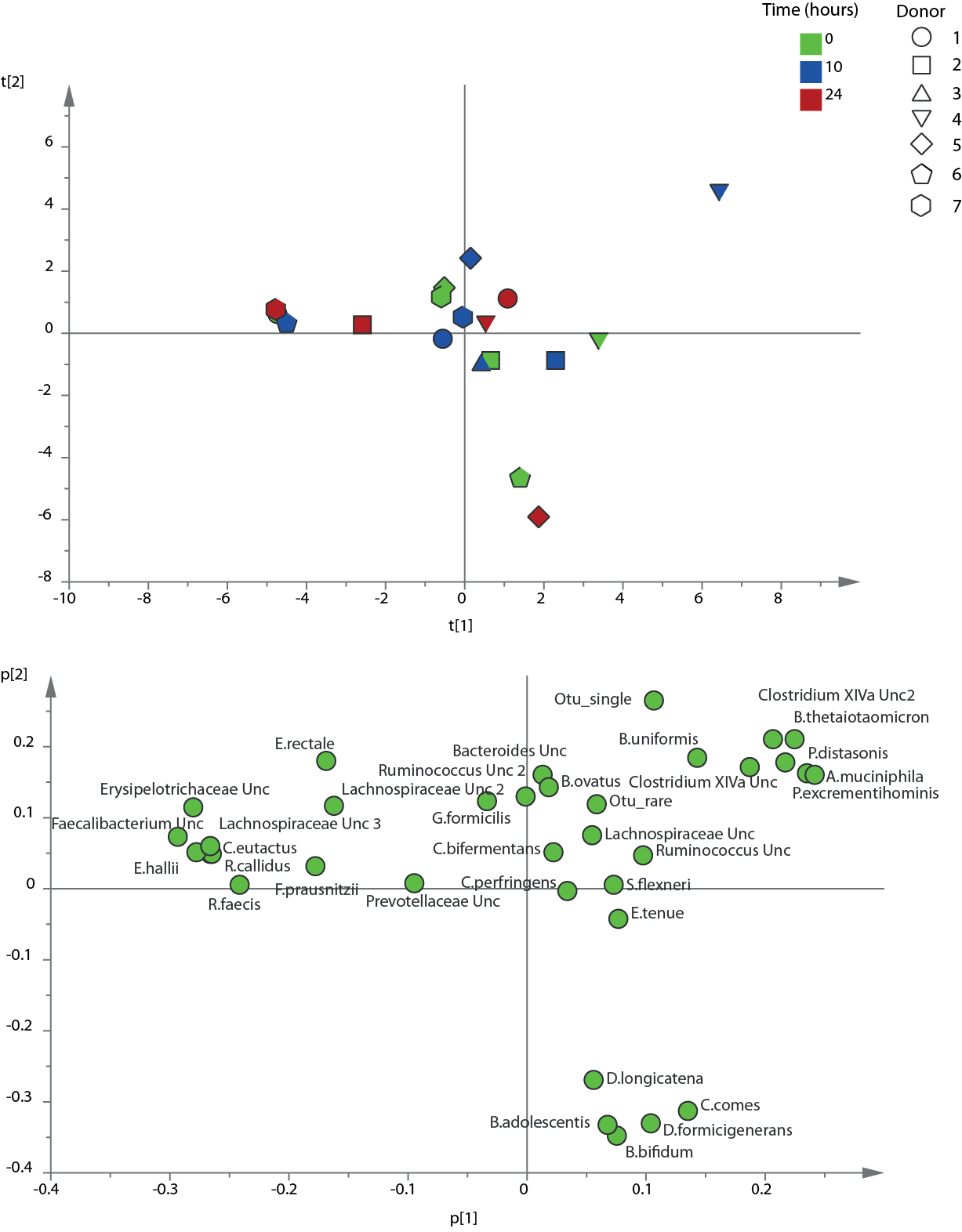


**Figure S4:** Scores (upper panel) and loadings (lower panel) plots derived from the principal components analysis (PCA) model built on the bacterial profiles of the individual donors receiving taurocholic acid across the study.
